# Improved Whole Gamma Irradiated Avian Influenza Subtype H9N2 Virus Vaccine Using Trehalose and Optimization of Vaccination Regime on Broiler Chicken

**DOI:** 10.3389/fvets.2022.907369

**Published:** 2022-07-12

**Authors:** Farahnaz Motamedi Sedeh, Iraj Khalili, Viskam Wijewardana, Hermann Unger, Parvin Shawrang, Mehdi Behgar, Sayed Morteza Moosavi, Arash Arbabi, Sayedeh Maede Hosseini

**Affiliations:** ^1^Nuclear Agriculture Research School, Nuclear Science and Technology Research Institute (NSTRI), Karaj, Iran; ^2^Quality Control Department, Razi Vaccine and Serum Research Institute (RVSRI), Agricultural Research Education and Extension Organization (AREEO), Karaj, Iran; ^3^Animal Production and Health Section, Department of Nuclear Sciences and Applications, Joint FAO/IAEA Centre of Nuclear Techniques in Food and Agriculture International Atomic Energy Agency (IAEA), Vienna, Austria; ^4^School of Medicine, Tehran University of Medical Science, Tehran, Iran

**Keywords:** avian influenza virus, gamma-radiation, vaccine, immune response, virus shedding

## Abstract

Gamma (γ)-radiation can target viral genome replication and preserve viral structural proteins compared to formalin inactivation. Thus, a stronger immunity could be induced after the inoculation of the irradiated virus. In this study, γ-irradiated low-pathogenic avian influenza virus-H9N2 (LPAIV-H9N2) was used to immunize the broiler chicken in two formulations, including γ-irradiated LPAIV-H9N2 with 20% Trehalose intranasally (IVT.IN) or γ-irradiated LPAIV-H9N2 plus Montanide oil adjuvant ISA70 subcutaneously (IV+ISA.SC) in comparison with formalin-inactivated LPAIV-H9N2 vaccine intranasally (FV.IN) or formalin-inactivated LPAIV-H9N2 plus ISA70 subcutaneously (FV+ISA.SC). Two vaccination regimes were employed; the first one was primed on day 1 and boosted on day 15 (early regime), and the second one was primed on day 11 and boosted on day 25 (late regime). A challenge test was performed with a live homologous subtype virus. Virus shedding was monitored by quantifying the viral load *via* RT-qPCR on tracheal and cloacal swabs. Hemagglutination inhibition (HI) antibody titration and stimulation index (SI) of the splenic lymphocyte proliferation were measured, respectively, by HI test and Cell Proliferation assay. Cytokine assay was conducted by the RT-qPCR on antigen-stimulated spleen cells. The results of the HI test showed significant increases in antibody titer in all vaccinated groups, but it was more evident in the IVT late vaccination regime, reaching 5.33 log_2_. The proliferation of stimulated spleen lymphocytes was upregulated more in the IVT.IN vaccine compared to other vaccines. The mRNA transcription levels of T-helper type 1 cytokines such as interferon-gamma (IFN-γ) and interleukin 2 (IL-2) were upregulated in all vaccinated groups at the late regime. Moreover, IL-6, a pro-inflammatory cytokine was upregulated as well. However, upregulation was more noticeable in the early vaccination than the late vaccination *(p*< *0.05)*. After the challenge, the monitoring of virus shedding for the H9 gene represented an extremely low viral load. The body weight loss was not significant (*p* > *0.05*) among the vaccinated groups. In addition, the viral load of <10^0.5^ TCID_50_/ml in the vaccinated chicken indicated the protective response for all the vaccines. Accordingly, the IVT vaccine is a good candidate for the immunization of broiler chicken *via* the intranasal route at late regime.

## Introduction

Among the three types of influenza viruses (A, B, and C), only influenza A genus has been isolated from birds and termed as avian influenza virus (LPAIV-H9N2). According to previous studies (1–3), influenza type A viruses are divided into subtypes based on the genetic and antigenic differences in two surface spike proteins, namely, hemagglutinin (HA) and neuraminidase (NA). The subtype LPAIV-H9N2 was initially isolated (1966) from turkeys in the northern state of the United States (4). Then, it was detected in domestic poultry in Europe, Africa, Asia, and the Middle East. Frequent outbreaks have been reported in Asian countries such as Iran, Saudi Arabia, Pakistan, and Iraq (2). After the first reported outbreak (1998), the virus became endemic, which led to a routine vaccination program to control LPAIV-H9N2 (1, 5). H9N2 is commonly co-circulating in poultry with other subtypes of LPAIV-H9N2, including H5 and H7 (6). Live poultry markets are a crucial link in the poultry trade and require close surveillance. H9N2 can be the donor of genes to other AIVs such as H5N1, H5N6, H7N9, and H10N8, which are responsible for high death rates in humans (7). In addition, H9N2 is a low-pathogenic Avian Influenza virus (LPAIV-H9N2) and has a wide host range such as ducks, chickens, pigs, and turkeys (8) with possible transmission from avian to humans (5). It causes considerable economic losses in the poultry industry worldwide (4). H9N2 infection leads to high economic losses in both layers and for breeders due to a drop in egg production. Broilers may also show severe losses during coinfection with other pathogens, especially Infectious Bronchitis Virus (IBV), Newcastle disease virus (NDV), bacteria such as E. coli and Mycoplasma, or even live virus vaccines (9). Therefore, a vaccine yielding a higher level of protection is required to prevent LPAI H9N2 in the poultry industry.

γ-Ray is ionizing radiation emitted from radio isotopes (Cobalt-60 and Cesium-137 isotopes) and high- or low-energy electron beams used for virus inactivation without any changes in viral proteins. The dose of γ-radiation for virus inactivation depends on the radiation temperature, the virus concentration, size of the viral genome, the presence of oxygen, and water content during the irradiation process (10). γ-Rays are at the higher frequency end of the electromagnetic spectrum (the shortest wavelength, but high energy). It is the perfect method for virus inactivation and destroys genetic materials by creating breaks in the genome (breaking ssRNA, dsRNA, or dsDNA).

The potency of γ-radiation has been successfully tested in human clinical trials for radiation-attenuated anti-parasite vaccines against malaria (11). For larger pathogens such as parasites and bacteria, a relatively low dose of γ-irradiation is sufficient for inactivating the organism (e.g., malaria irradiation at 150 Gray, Fasciola irradiation at 30 Gray, and Brucella irradiation at 6 kGy). Conversely, viral pathogens require higher doses, including Rift Valley Fever virus irradiation at 25 kGy (12, 13), LPAIV-H9N2 at 30 kGy (14), foot and mouth disease virus at 45 kGy (15), and poliovirus subtypes 1 and 3 at 35 kGy (16).

The use of these higher doses of irradiation for virus inactivation takes a longer time and results in building up free radicals that could damage the antigenic epitopes of viral proteins, even if that could be lesser extent compared to chemical inactivation (11–15). Although many compounds could be used to rescue this damage which is caused by the free radicals. Trehalose, as a disaccharide, has its own merits as a cryo-protect and a free radical quencher (17–19). Trehalose can stabilize proteins and inhibit protein denaturation by excluding water molecules from the surface of proteins when cells are in a dehydrated condition. The dry state maintains proteins in the folded state by replacing water molecules and forming hydrogen bonds directly with proteins and thus their structure. Trehalose acts as a natural stabilizer of life processes (17–19).

In this research, the LPAIV-H9N2 was irradiated after formulation with Trehalose and employed to immunize the broiler chicken in two formulations and at two vaccination regimes *via* two routes of administration. Virus shedding and immune responses were evaluated for 48 days. The specific objectives of this study are to use Trehalose as a protein stabilizer during LPAIV-H9N2 irradiation, use of irradiated LPAIV-H9N2 plus Trehalose or Montanide ISA70 as an inactivated vaccine, comparison of two vaccination regimes *via* two routes of administration, early and late regimes, vaccine inoculation subcutaneously or intranasal for evaluating immune responses due to irradiated LPAIV-H9N2 vaccine and formalin LPAIV-H9N2 vaccine and comparison of immune responses between irradiated LPAIV-H9N2 vaccine and formalin-inactivated LPAIV-H9N2 vaccine. Furthermore, we can suggest the more protective vaccine, the better route of administration and vaccination regime against LPAIV-H9N2.

## Materials and Methods

### Vaccine Preparation

The isolated LPAIV-H9N2 strain A/chicken/IRN/Ghazvin/2001 was a kind donation from the Razi Vaccine and Serum Research Institute of Iran. The irradiated avian influenza subtype H9N2 vaccine was prepared according to the protocols published by Javan et al. (14) and Salehi et al. (20). Briefly, 3–4 days after the multiplication of the LPAIV-H9N2 on embryonated specific free pathogen (SPF) chicken eggs, the allantoic fluid was collected and tested using a hemagglutination test for HA titration. The infected allantoic fluid was used for virus titration measuring embryo infective dose (EID50) and calculated according to the formula by Reed and Muench (21). A γ-ray dose of 30 kGy was recommended for the frozen virus suspension (22). Half of the frozen LPAIV-H9N2 stock plus 20% Trehalose (1 M, a disaccharide of glucose and as a protein protectant) and half of the frozen LPAIV-H9N2 stock without Trehalose were irradiated using the gamma irradiator (Nordion Company, Canada, model 220, γ-cell) at a dose rate of 2.07 Gy/s and activity of 8677 Ci for virus inactivation on dry ice. The Laemmli SDS-PAGE system was used to assess the quality of viral proteins in irradiated and non-irradiated viral samples. In this study, the γ-irradiated LPAIV-H9N2 was applied in two formulations. The first one was γ-irradiated LPAIV-H9N2 with 20% disaccharide Trehalose (1 M) as a water suspension (IVT vaccine) and the second one was γ-irradiated LPAIV-H9N2 plus Montanide oil (ISA70) as a water-in-oil (30/70) vaccine (IV+ISA vaccine). A formalin concentration of 0.1% at 25 °C was added to the LPAIV-H9N2 suspension to inactivate the virus for 24 h and employed as the formalin-treated vaccine (FV) according to Razi protocols (20, 22). The FV was used in two formulations. The first one was used as a drop on the nose (FV.IN) by 10^7.5^ EID_50_/100 μl and the second one was an FV plus Montanide oil (ISA70) as a water-in-oil (30/70) vaccine subcutaneously (FV+ISA.SC vaccine). Vaccination was performed in two routes of administration (intranasally and subcutaneously injection on the neck) on broiler chicken. In addition, Montanide ISA 70 was applied as an adjuvant, along with irradiated and formalin vaccines. Injectable vaccine and stable water-in-oil (W/O) emulsions were obtained by mixing Montanide ISA70 and antigenic media (H9N2) under a high shear rate.

### Animal Trails

The animal experiments were performed in two steps. The first chicken experiment was conducted on seven chicken groups. A total of forty-two broiler chickens (ROSS 308) were purchased from Alborz hatchery center and allocated into seven groups, each including 6 animals (three chickens were used in each of sampling). The first group was pre-immunization and the second group was the negative control group and was inoculated with sterile PBS intranasally. The third and fourth groups were immunized by irradiated vaccine intranasally or subcutaneously (IV.IN or IV.SC). Moreover, the fifth and sixth groups were vaccinated by irradiated vaccine plus 20% Trehalose intranasally or subcutaneously (IVT.IN or IVT.SC), and the seventh group was inoculated by Trehalose solution (1 M) alone intranasally (T.IN). The amount of each vaccination dose was 100 μl. The sera samples were collected from all chickens 2 weeks after the first and second vaccination for HI antibody titration assay (at days 25 and 38). The splenic lymphocytes of all chicken groups were cultured and stimulated by homologous-inactivated antigens to evaluate the splenic lymphocyte proliferation assay as cellular immunity at days 25 and 38 (three chickens in each group were used for each sampling day).

**Figure 1:**
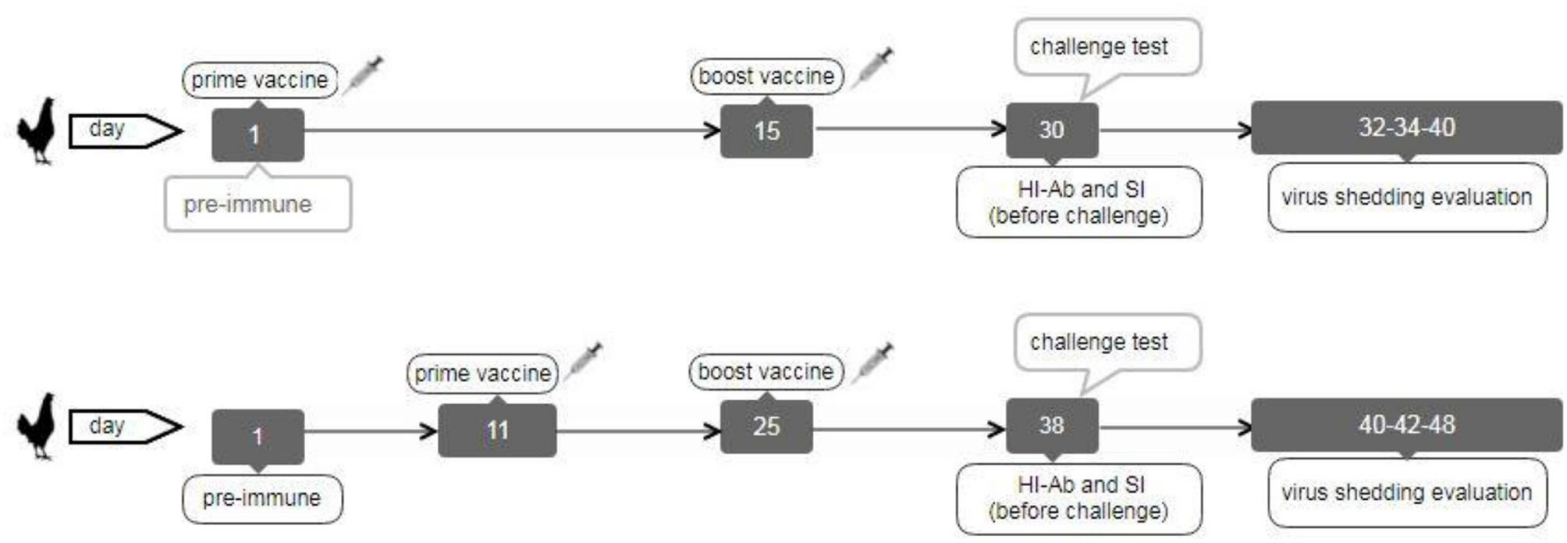
The broiler chicken vaccination diagram in early (top) and late (down) regimes.

The second chicken experiment was performed on the other vaccinated bird groups (Table 1). A total of 200 and21 broiler chickens (ROSS 308) were purchased and allocated into 17 groups (each including 13 chickens) to evaluate immune responses and virus shedding in the second animal experiment. The first group was pre-immunization, and the second and third groups were inoculated with sterile PBS intranasally or subcutaneously as negative control groups (NC.IN or NC.SC). Further, the fourth to eighth groups were vaccinated with irradiated LPAIV-H9N2 plus Trehalose and the two routes of administration, namely intranasally (IVT.IN) or subcutaneously (IVT.SC), formalin LPAIV-H9N2 intranasally (FV.IN), formalin LPAIV-H9N2 plus ISA70 subcutaneously (FV+ISA.SC), irradiated LPAIV-H9N2 plus ISA70 subcutaneously (IV+ISA.SC), respectively. The vaccination was conducted in two different regimes (Figure 1). The first one was primed on day 1 and boosted on day 15 (early regime), and the second one was primed on day 11 and boosted on day 25 (late regime); one hundred and four chickens were used in each regime. The second to eighth groups and the ninth to fifteen groups were vaccinated in the early and late regimens, but the same vaccines. The last two groups (16 and 17 groups) were positive control, without vaccination and challenged with live homologous subtype virus (Table 1). The sera samples were collected from all chickens 2 weeks after the second vaccination and before challenge with live virus (at days 30 and 38 for early and late regimes, respectively) for HI antibody titration assay. The splenic lymphocytes of the chicken groups (three chickens in each sampling day, 84 chickens in early and late regimens groups, and 13 chickens in pre-immune group, totally 97 chickens were used for culturing splenic lymphocyte) were cultured and stimulated by homologous-inactivated antigens to measure the splenic lymphocyte proliferation assay as cellular immunity, and cytokines assay at days 30 (before challenge with live virus) and 40 in early regime and at days 38 (before challenge with live virus) and 48 in late regime, respectively.

**Table 1:** The second design for the chicken experiment.

**No**	**Vaccine groups**	**Route- of Adm**	**Vaccine dose (μl)**	**Vaccination day**	**Regime of Vaccination**	**Challenge-day**	**Number of chicken**	**Sampling day for HI antibody titration**	**Sampling days for SLP assay & cytokine assay / number of chicken**	**Sampling days after challenge for virus shedding**	**Sampling days after challenge for virus shedding in lung tissue / Number of chicken**
1	Pre-Immune	-	-	-	-	-	13	1	1/13	-	-
2	N.C (PBS)	IN	100	1,15	early	-	13	30	30/3 40/3	2, 4, 10	5/3
3	N.C (PBS)	SC	100	1,15	early	-	13	30	30/3 40/3	2, 4, 10	5/3
4	IVT	IN	100	1, 15	early	30	13	30	30/3 40/3	2, 4, 10	5/3
5	FV	IN	100	1,15	early	30	13	30	30/3 40/3	2, 4, 10	5/3
6	IVT	SC	100	1,15	early	30	13	30	30/3 40/3	2, 4, 10	5/3
7	FV+ISA	SC	100	1,15	early	30	13	30	30/3 40/3	2, 4, 10	5/3
8	IV+ISA	SC	100	1,15	early	30	13	30	30/3 40/3	2, 4, 10	5/3
9	N.C (PBS)	IN	100	11,25	late	-	13	38	38 /3 48/3	2, 4, 10	5/3
10	N.C (PBS)	SC	100	11,25	late	-	13	38	38/3 48/3	2, 4, 10	5/3
11	IVT	IN	100	11, 25	late	38	13	38	38/3 48/3	2, 4, 10	5/3
12	FV	IN	100	11, 25	late	38	13	38	38/3 48/3	2, 4, 10	5/3
13	IVT	SC	100	11, 25	late	38	13	38	38/3 48/3	2, 4, 10	5/3
14	FV+ISA	SC	100	11, 25	late	38	13	38	38/3 48/3	2, 4, 10	5/3
15	IV+ISA	SC	100	11, 25	late	38	13	38	38/3 48/3	2, 4, 10	5/3
16	PC	IN	-	-	-	30	13	-	-	2, 4, 10	5/3
17	PC	IN	-	-	-	38	13	-	-	2, 4, 10	5/3

*N.C, negative control; IV, Irradiated Vaccine; IVT, Irradiated Vaccine + Trehalose; IN, Intranasal; SC, Subcutaneous; Adm, Administration; SLP, Splenic Lymphocyte Proliferation Assay; FV, Formalin Vaccine; (FV+ISA), Formalin Vaccine plus Montanide oil ISA70; (IV+ISA), Irradiated Vaccine plus Montanide oil ISA70*.

### Challenge of Vaccinated Chicken With the Live Homologous Virus and Virus Shedding

The challenge test with live homologous virus was done in the poultry isolators with Hepa Filter in the biosafety level 2+ laboratory (in Iran Veterinary Organization (IVO), National Diagnosis Center, Reference Laboratories & Applied Studies). All the vaccinated chicken groups in the second animal experiment were challenged with 50 μl live homologous subtype virus (10 ^5.5^ EID_50_/ml) through intranasal and ocular routes, except for pre-immunization and negative control groups (1, 2, 3, 9, and 10 groups), including the five early groups on day 30 and the five late groups on day 38 (ten chickens remained in each group for challenge). Furthermore, two positive control groups (each including 13 chickens) were inoculated with 50 μl live homologous subtype virus (10 ^5.5^ EID_50_/ ml) *via* intranasal and ocular routes at days 30 and 38 days. Tracheal and cloacal swabs were collected and used for RNA extraction on 2, 4, and 10 days after the challenge from the vaccinated bird groups, negative control and positive control groups to evaluate virus shedding by the RT-qPCR.

Morbidity and body weight loss for all chicken groups were daily monitored for 10 days following the challenge. Similarly, the lung tissue of three chickens in each group was harvested and homogenized 5 days after the challenge, and then the virus titer was determined using the Madin-Darby canine kidney (MDCK) cells and Hemagglutinin antigen assay (HA assay). To release the virus, the lung tissue homogenates were frozen and thawed three times, and their supernatants, in serial dilution from 10^−1^ to 10^−7^-fold, were added to triplicate the wells of MDCK cells. After incubation for 1 h, RPMI 1640+ 10% FCS was added to each well and incubated for 48 h at 37 °C in a humidified incubator (New Brunswick, England) containing 5% CO_2_. HA was performed on the supernatant of each well by the co-incubation of the culture supernatant with chicken red blood cells. The virus titration in lung tissues was determined by interpolating the dilution endpoint that infected the cells in 50% of the wells and as log_10_ TCID_50_ (Tissue Culture Infectious Dose 50%).

### Evaluation of Immune Responses and Cytokine Assay

The diluted chicken sera (two-fold serially diluted with sterile PBS) were mixed with four hemagglutinin units of virus antigens (the infected allantoic fluids) in 96-well microplates and incubated 30 min at room temperature (23). Chicken red blood cells (0.5%) were added to the mixtures and set for 30 min at room temperature. The reciprocals of the highest serum dilutions showing complete HI were expressed as the HI titration. As explained in Table 1 for the second chicken experiment, the cellular immunity was measured by the splenic lymphocyte proliferation (SLP) assay at days 30 and 38 for five vaccinated chicken groups and two negative control groups in the early regime, the other five vaccinated chicken groups and two negative control groups in the late regime, respectively (24–26). Briefly, the spleens of the immunized chickens were aseptically removed 2 weeks after the boost immunization (at days 30 and 38), and single splenic lymphocyte suspensions were prepared (25, 26) and incubated in 96-well plates at a density of 2 × 10^5^ cells/well by RPMI 1,640 (Invitrogen, USA) + 10% fetal calf serum (FCS) (ZiSera, Iran) at 37 °C in an incubator with humidified atmosphere containing 5% CO_2_. The cells were stimulated by irradiated inactivated homologous LPAIV-H9N2 (3 μl/well) as the specific antigen for the vaccine groups, as well as phytohemagglutinin (5 μg/ml) for the positive control, or without any stimulating antigen in triplicates. Then, 48 h post-stimulation, the supernatant of splenic cells was collected for cell proliferation assay according to the protocol of the cell proliferation MTT kit (Roche, England). The Cell Proliferation MTT Kit is a colorimetric assay for the nonradioactive quantification of cellular proliferation. The tetrazolium salt (MTT) is cleaved to formazan by enzymes of the endoplasmic reticulum. This bio-reduction occurs in viable cells only, and is related to NAD(P)H production through glycolysis. The MTT solution was added (30 μl) per well with a concentration of 5 mg/ml. After 4 h of incubation at 37 °C, 100 μl of dimethyl sulfoxide was added to each well to dissolve formazan crystals. The optical density was measured at 540 nm. Finally, the stimulation index (SI) was calculated for each sample (SI = mean of optical density for stimulated wells/mean of OD unstimulated wells). Additionally, the pellet of splenic cells was collected and suspended in Trizol solution for RNA extraction to assess interleukin 2 (IL-2), IL-6, and interferon-gamma (IFN-γ) production by the real-time polymerase chain reaction (RT-PCR). RNA was extracted by the RNA Mini Kit (Bio&Sell, Germany). The concentration of RNA was measured by the Nanodrop system (Smart, Canada). Then, cDNA was synthesized by the Easy cDNA synthesis kit (Parstous, Iran, Cat A101161). Briefly, the template RNA (1 ng−5 μg), buffer mix-2X (10 μl), enzyme mix (2 μl), and DEPC water (up to 20 μl) were mixed in an RNase- free tube. The above mixture was mixed by the quick vortex, incubated 10 and 60 min at 25 and 47 °C, respectively, stopped the reaction by heating at 85 °C for 5 min, and finally, chilled at 4 °C. According to the manufacturer's protocol, the synthesized cDNA was used for the real-time RT-PCR by IQ SYBR Green Supermix (Bio-Rad, Cat.No.172-5270, United State) at SYBR/FAM channel with a Rotor Gene-Q (Qiagen, German) real-time PCR system. Real-time PCR reactions were set up at 95 °C for 3 min, 40 cycles at 95 °C for 10 s, 59 °C for 20 s, and 72 °C for 20 s. The cycle threshold (Ct) of gene products was determined and set to the log-linear range of the amplification curve and kept constant. The relative expression of cytokines was calculated as the fold change (2^−Δ*ΔCt*^) with normalization to the corresponding GAPDH values as the housekeeping gene used in this study (25, 26).

### RNA Extraction and cDNA Synthesis

The tracheal and cloacal swabs of vaccinated chicken in the second animal experiment were collected 14 days after the second vaccination and after challenge with live virus in RNX-Plus solution (Sinaclon Iran). The RNA was extracted by the RNA Mini Kit (Bio & Sell, Germany). Briefly, the swab samples were lysed by 400 μl of the lysis solution (in the RNA Mini kit), clarified on Spin Filter E, and centrifuged at 10,000 × g for two min. The sample was bound on Spin Filter S and washed by the washing solution. Finally, the column was dried, and RNA was eluted in RNase-free water. According to the manufacturer's protocol, the cDNA was synthesized using SCRIPTUM first-strand cDNA synthesis for efficient reverse transcription (Bio & Sell, Germany). The concentration of RNA was measured by the Nanodrop system (Smart, Canada). The specific primer for the HA gene (H9) was used according to Ong et al. (27). Briefly, 50 pg of total RNA, 20 pg of the forward specific primer for the H9 gene (5‘-CTACTGTTGGGAGGAAGAGAATGGT-3‘), and 20 μl RNase free water were mixed, followed by adding dNTP Mix (5 mM, 1 μl), RT buffer (1X, 4 μl), DTT stock (5 mM, 1 μl), and SCRIPTUM first reverse transcriptase (100 units) and incubating them at 50°C for 30–60 min. The cDNA could now be applied as a template in PCR or stored at −20°C (28). The concentration of cDNA was measured by the Nanodrop system (Smart, Canada).

### Conventional RT-PCR and Real-Time Reverse Transcription (RT)-PCR for the H9 Gene

The extracted RNA was reverse transcribed and amplified for conventional RT-PCR using the H9 gene-specific primer pair (F: 5‘-CTACTGTTGGGAGGAAGAGAATGGT-3‘ and R: 5‘-TGGGCGTCTTGAATAGGGTAA-3‘) and AccuPower one-step RT-PCR kit (Bioneer, Daejeon, Korea) (27). Then, the HA gene was deposited with accession number FJ794817, and the PCR products for positive samples were detected as a band with 256 bp in size by agarose gel (1.2%) electrophoresis. The extracted RNA of the LPAIV-H9N2 isolate H9N2/A/chicken/IRN/Ghazvin/2001 (FJ794817) at a viral load of 10 ^8.5^ EID_50_/ml was used to compare the limit of detection by real-time RT-qPCR and conventional RT-PCR. The conventional RT-PCR and real-time RT-qPCR results for qualification of the H9 gene were compared using the serial dilutions of cDNA (with H9 specific primers), and cDNA concentration was measured by the Nanodrop system (Smart Nano, Canada) (28, 29). Moreover, according to the manufacturer's protocol, the cDNA of all samples for virus gene (H9) quantitation was amplified using QPCR Mix EvaGreen kit (Bio & Sell, Germany). The reaction mixture contained 100 nM of each specific primer pair for the H9 gene, which was used for conventional PCR, and for RT-QPCR we used 5 × QPCR mix EvaGreen (4 μl), cDNA as a template (5 μl) and up to 20 μl water (30). The reaction was performed at the initial incubation temperature (94°C) for 3 min, and then 40 three-step cycles (30 s at 94°C for denaturation, 30 s at 58°C for annealing, and 30 s at 72°C for elongation) by the Rotor-Gene Q (QIAGEN) system.

### Statistical Analysis

All statistical analyses were conducted by one-way ANOVA and Duncan's multiple range test. The comparisons of means for the antibody titration and SI in the first chicken experiment with sample size: 42 chickens, and the comparisons of means for the antibody titration, SI and cytokines values in the second chicken experiment with sample size: 200 and 21 broiler chickens, were done using SPSS, version 16. All values were expressed as the mean ± standard deviation, and a *p* ≤ *0.05* was considered statistically significant.

## Results

**Figure 2:**
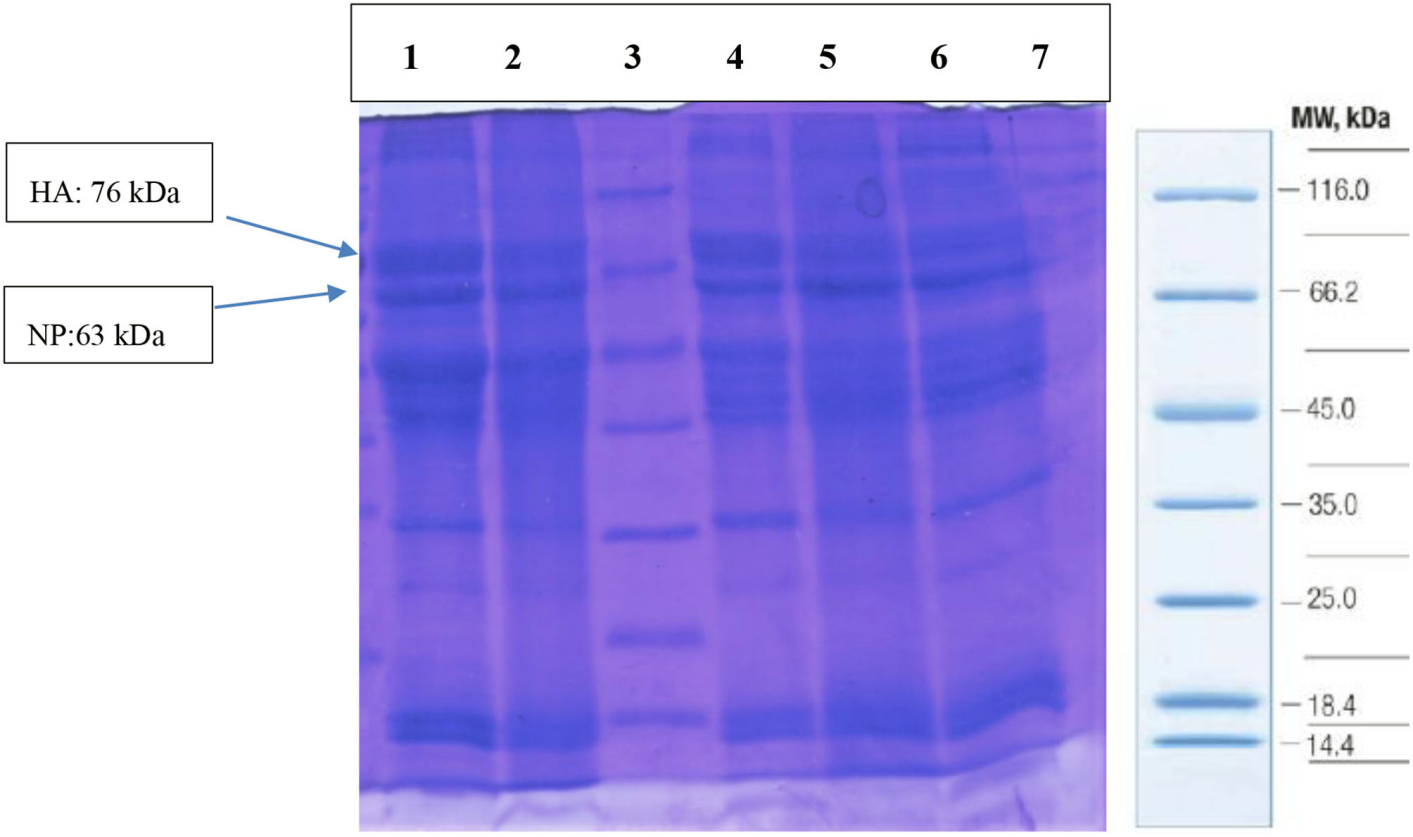
The quality of viral protein by SDS-PAGE in irradiated (Lanes: 4, 5, 6) and non-irradiated (Lanes: 1, 2) LPAIV-H9N2 samples. Lane 3: protein markers (Cat No: SM0431), HA, Hemagglutinin antigen; NP, Nucleoprotein.

The virus titer was 10^8.5^ EID_50_/ml, and the HA assay for irradiated and non-irradiated avian influenza A subtype H9N2 virus samples was 10 Log_2_, suggesting that irradiation does not lead to a decrease in the HA antigen. The D_10_ value and inactivation dose of γ-radiation were 3.4 and 30 kGy, respectively. The safety test was performed for irradiated LPAIV-H9N2 (at 30 kGy) on SPF eggs by four blind cultures and represented no virus multiplication at 30 kGy (19, 29). Also, the SDS-PAGE electrophoresis showed no change in the viral protein bands (HA and NP proteins) in irradiated and non-irradiated viral samples (Figure 2).

**Figure 3:**
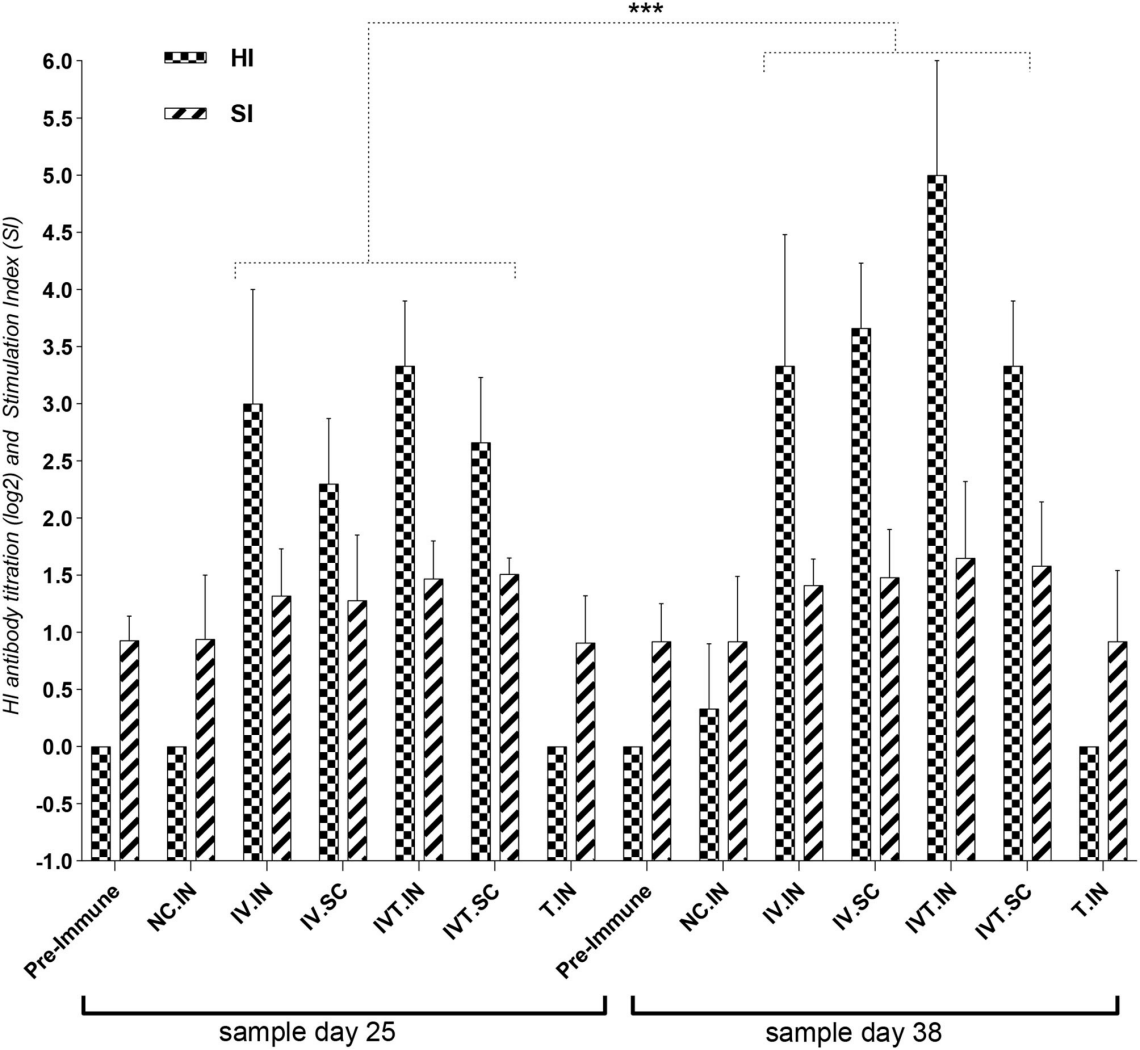
The first design for the chicken experiment, evaluation of HI antibody titration and Stimulation Index (SI) for Splenic Lymphocyte Proliferation Assay for vaccinated chicken groups. N.C, negative control; IV, Irradiated Vaccine; IVT, Irradiated Vaccine + Trehalose; IN, Intranasal; SC, Subcutaneous; Adm, Administration. ***P ≤ 0.05.

The data in the first chicken experiment (Figure 3) showed a significant increase in IVT.IN group for HI antibody titration and the proliferation index of stimulated spleen lymphocytes in IVT.IN and IVT.SC groups (*p*< *0.05*). The first chicken experiment showed the increasing of the HI antibody titration and stimulation index of stimulated spleen lymphocytes 34 and 15%, in IVT.IN compered to IV.IN vaccine group, respectively.

**Figure 4:**
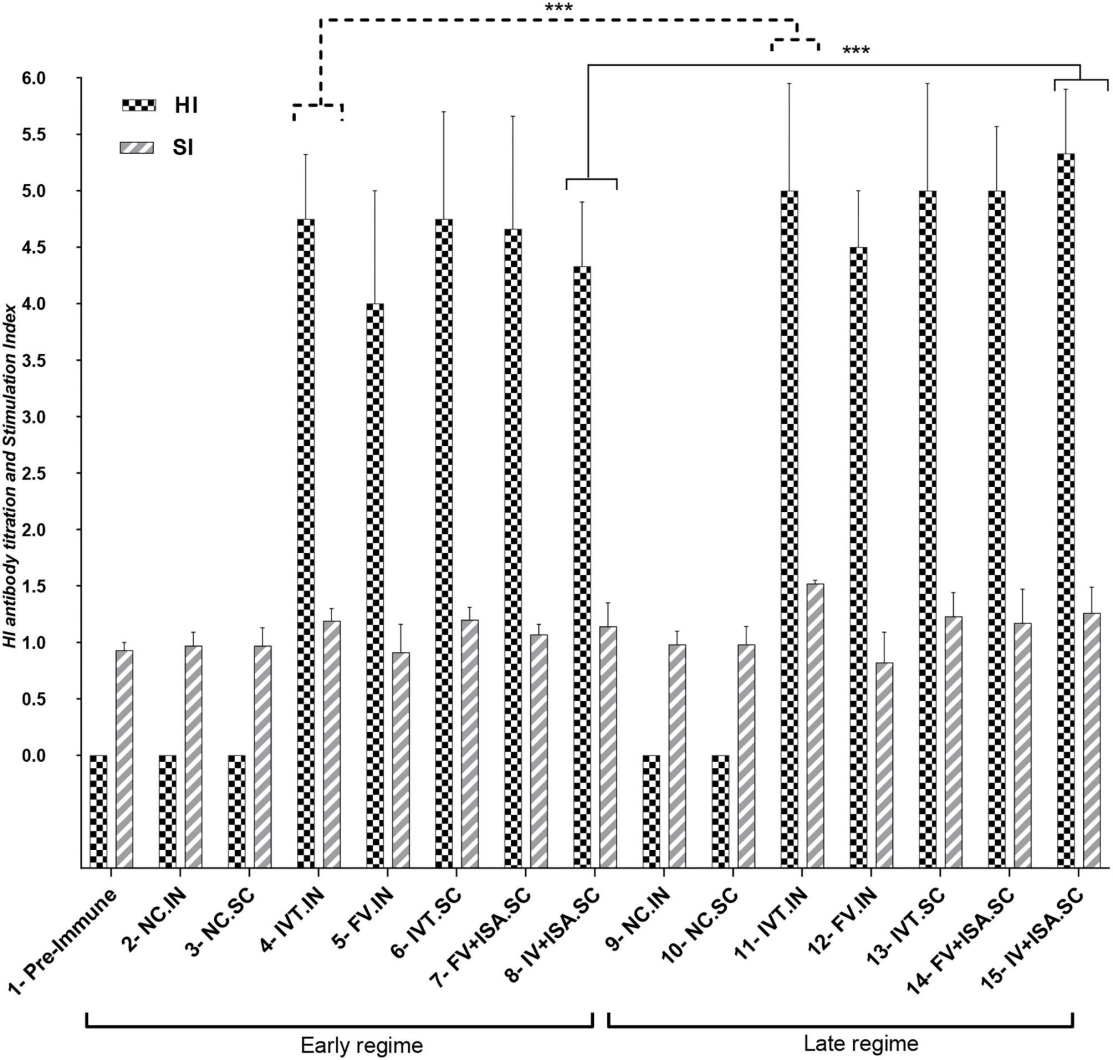
HI antibody titration and Stimulation Index for Splenic Lymphocyte Proliferation Assay for vaccinated chicken groups in the second animal experiment (early regime vaccinated at days 1 and 15, Late regime vaccinated at days 11 and 25), the sampling was done on 30 and 38 days. N.C, negative control; IVT, Irradiated Vaccine + Trehalose; FV, formalin Vaccine; IN, Intranasal; SC, Subcutaneous (^***^p ≤ 0.05).

The data in the second chicken experiment showed a significant increase in HI antibody titration in all vaccinated bird groups relative to the negative control (*p*< *0.05*), but the most significant increase was observed in the IVT.IN vaccine and the IV+ISA vaccine (SC) groups (*p*< *0.05*). The comparison of the antibody titration between two vaccination regimes demonstrated more HI titers in the late-vaccinated groups reaching up to 5.33 log_2_ in 2 weeks after the second vaccination. However, these levels were not significantly different in other groups (Figure 4).

**Figure 5:**
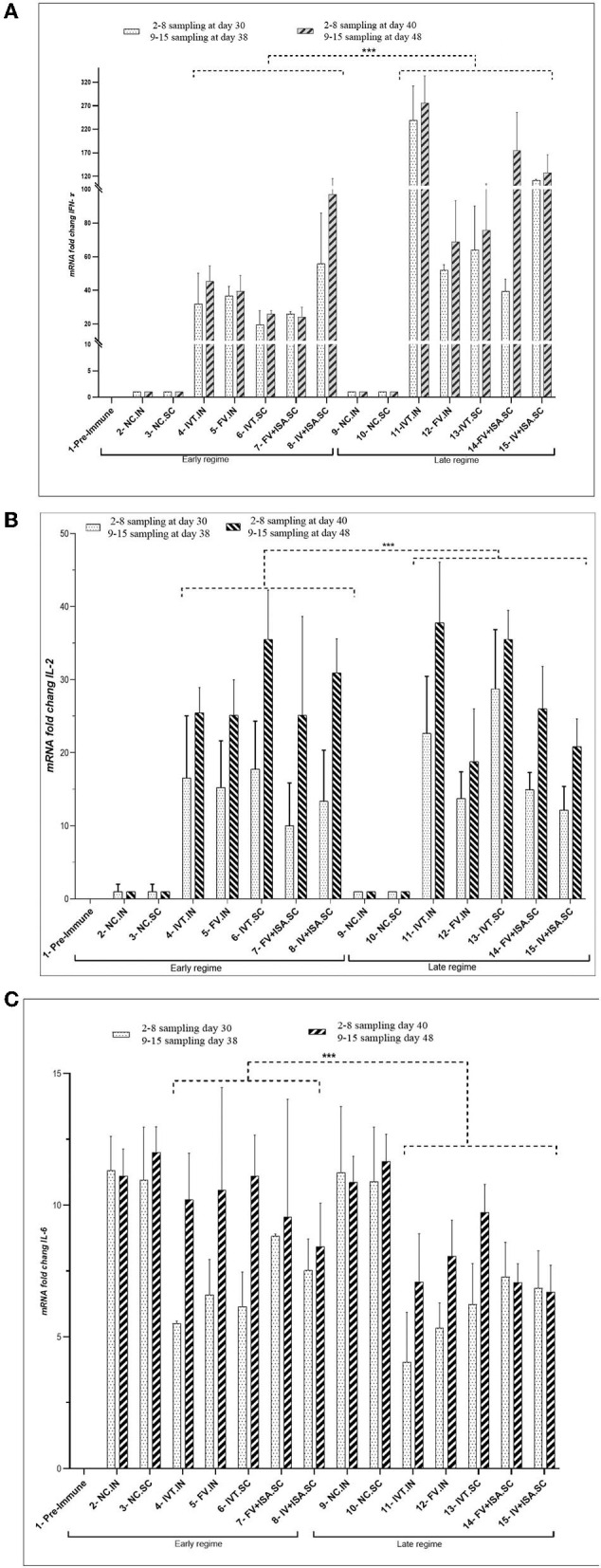
The cytokine assay **(A)** IFN-γ, **(B)** IL-2, and **(C)** IL-6 (cytokines mRNA transcription levels as fold change) in the vaccinated broiler chicken groups in the second animal experiment). Two vaccination regimes were performed: the early regime was primed on day 1 and boosted on day 15 (early regime) and sampling was done on 30 and 40 days. The late regime was primed on day 11 and boosted on day 25 (late regime) and sampling was done on 38 and 48 days. N.C, negative control; IVT, Irradiated Vaccine + Trehalose; FV, formalin Vaccine; IN, Intranasal; SC, Subcutaneous (^***^p ≤ 0.05).

The proliferation index of stimulated spleen lymphocytes was significantly upregulated in all vaccinated chicken groups in the second chicken experiment (*p*< *0.05*). However, the most upregulation was detected in the IVT.IN and the IV+ISA *(p*< *0.05*) vaccines in the late vaccinated regime. Based on these data, the cellular immunity induction in the irradiated vaccine groups was more considerable compared to formalin-treated vaccine groups (Figure 4). In addition, cytokines mRNA expression was upregulated in all vaccinated groups, and T-helper type 1 (Th1) cytokines such as IFN-γ and IL-2 were expressed more in the late vaccinated chicken regime. In this study, IL-6 was upregulated in all vaccinated chicken groups. However, upregulation was more noticeable in the early vaccination than the late vaccination *(p*< *0.05)* (Figure 5). However, IL-6 was down-regulated in all vaccinated chicken groups in early and late regimes before the challenge with the live virus compared to these groups after the challenge.

### The Conventional and Real-Time RT-qPCR Results for the H9 Gene

**Figure 6:**
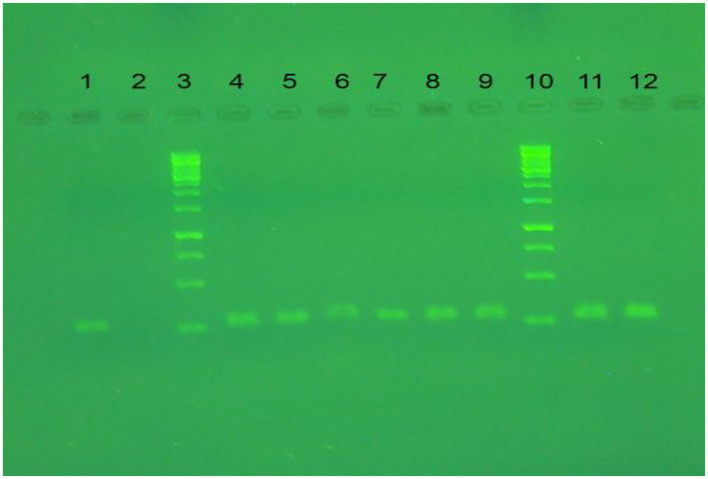
The result of conventional RT-PCR on agarose gel. Lanes 1, 11, and 12 are RT-PCR products of LPAIV-H9N2 *via* specific primer for H9 gene. Lanes 3 and 10 are DNA ladder (SM0313). Lane 2 is a negative control, lanes 4-9 are RT-QPCR products for H9N2 cDNA dilution (10^−1^-10^−3^) in duplication.

The extracted RNA of the Avian Influenza virus isolate H9N2/A/chicken/IRN/Ghazvin/2001 (FJ794817) at a viral load of 10 ^8.5^ EID_50_/ml was used to compare the limit of detection by real-time RT-qPCR and conventional RT-PCR (28). The conventional RT-PCR and real-time RT-qPCR results for qualification of the H9 gene were compared using the serial dilutions of cDNA (with H9 specific primers), and cDNA concentration was measured by the Nanodrop system (Smart Nano, Canada) and it was from 9,681 −0.05 ng/μl for 10^0^-10^−5^ dilutions. The detection limit for the H9 gene in the infected allantoic fluid was determined as 1.5 and 127 ng/μl by real-time RT-QPCR (A Ct value of about 27) and conventional RT-PCR (Figure 6), respectively.

The monitoring of virus shedding for the H9 gene at 2, 4, and 10 days after the challenge revealed no viral load in the tracheal samples (Table 2). The result of cloacal swab samples was the same as that of the tracheal samples. The Ct value for all tracheal and cloacal swab samples in groups 4–8 (early regime) and 11–15 groups (late regime) was more than 27 and negative for virus shedding after challenge. Virus shedding was not observed after challenging the vaccinated chickens with the live virus in tracheal and cloacal swab samples. It indicates that the immunization with irradiated LPAIV-H9N2 and formalin LPAIV-H9N2 can induce a protective response against the live homologous virus.

**Table 2:** The monitoring of virus shedding in vaccinated chickens in the second animal experiment at 2, 4, and 10 days after challenge with homologs subtype virus at tracheal and cloacal swab samples.

**No**	**Vaccine groups**	**Route- of Adm**	**Regime of Vaccination**	**Challenge-day**	**Sampling days after challenge**		
					**2**	**4**	**10**
					**Virus shedding CT (log10** ***)***		
1	Pre-Immune	-	-	-	-	-	-
2	N.C (PBS)	IN	Early	-	UND	UND	UND
3	N.C (PBS)	SC	Early	-	UND	UND	UND
4	IVT	IN	Early	30	1.543	1.535	1.525
5	FV	IN	Early	30	1.559	1.577	1.485
6	IVT	SC	Early	30	1.532	1.600	1.518
7	FV+ISA	SC	Early	30	1.582	1.549	1.521
8	IV+ISA	SC	Early	30	1.546	1.575	1.527
9	N.C (PBS)	IN	Late	-	UND	UND	UND
10	N.C (PBS)	SC	Late	-	UND	UND	UND
11	IVT	IN	Late	38	1.488	1.487	1.534
12	FV	IN	Late	38	1.492	1.483	1.563
13	IVT	SC	Late	38	1.512	1.554	1.565
14	FV+ISA	SC	Late	38	1.483	1.544	1.549
15	IV+ISA	SC	Late	38	1.512	1.544	1.561
16	PC	IN	-	30	1.308	1.193	UT
17	PC	IN	-	38	1.269	1.209	UT

*UND, Undetermined; UT, Un-tested*.

### Viral Load on Lung Tissues and Loss of Body Weight Post-Challenge

The HI antibody titer and SI of splenic lymphocyte proliferation were significantly enhanced in vaccinated chicken groups, especially in the late vaccinated IVT.IN group. To evaluate the clinical protective effect of vaccines, the vaccinated chicken was challenged by the intranasal route on day 14 after the second immunization and monitored for 10 days for body weight loss. According to Table 3, the percent of body weight gain in early regime vaccinated groups during 1–30 days was more than 82% and in late regime vaccinated groups during 11–38 days was more than 84%. As expected, all the chickens in positive control groups (without vaccination) showed rapid body weight loss 10 days after the challenge. The percent of body weight gain in the positive control groups was nearly 89% before the challenge (during 1–30 days and 11–38 days), and 10 days after the challenge, it decreased to 75%. This data on increasing body weight showed the efficacy of vaccines (Table 3). The viral load on lung tissues was assayed 5 days after the challenge. However, body weight loss was not significant among the vaccinated chicken groups *(p*>*0.05)*. The viral load in lung tissues was calculated by Reed and Munch's method as TCID_50_/ml. The viral load value in positive control groups was approximately 10^5^ TCID_50_/ ml. This value was <10 ^0.5^ TCID_50_/ ml in the negative control groups (without vaccination and not challenged with live homologous subtype virus) and all vaccinated chicken groups.

**Table 3:** The % increasing of body weight in early regime vaccinated groups during 1–30 days and in late regime vaccinated groups during 11–38 days.

**No**	**Vaccine groups**	**Route- of Adm**	**Regime of Vaccination**	**Challenge-day**	**% Increasing of body weight 1–30 and 11–38 days**	**% Increasing of body weight 1–40 and 11–48 days**
1	Pre-Immune	-	-	-	-	-
2	N.C (PBS)	IN	Early	-	89.14	90.14
3	N.C (PBS)	SC	Early	-	88.25	90.33
4	IVT	IN	Early	30	89.52	90.56
5	FV	IN	Early	30	87.26	88.76
6	IVT	SC	Early	30	88.41	89.38
7	FV+ISA	SC	Early	30	82.74	83.33
8	IV+ISA	SC	Early	30	84.55	85.11
9	N.C (PBS)	IN	Late	-	90.52	90.52
10	N.C (PBS)	SC	Late	-	88.75	90.35
11	IVT	IN	Late	38	89.58	91.03
12	FV	IN	Late	38	88.72	89.20
13	IVT	SC	Late	38	89.65	89.65
14	FV+ISA	SC	Late	38	84.20	84.96
15	IV+ISA	SC	Late	38	85.02	86.43
16	PC	IN	-	30	88.94	75.88
17	PC	IN	-	38	88.38	75.14

## Discussion

LPAIV-H9N2 usually causes clinical diseases, including generalized infections, upper respiratory disease, and decreased egg production in the hosts, such as the layer chicken (31–33). In addition, this virus causes specific viral diseases in many bird species. It is also one of the instances of human health risks because it can create disease by contact with infected poultry or meats in humans. Hemagglutinin subtypes H5, H7, and H9 are the most important viruses that may infect humans (33). H9N2 has widely circulated in the poultry population and caused economic losses (34, 35). This virus has low pathogenicity to birds, but it is a severe threat to public health (36). Although prevailing vaccines decrease the disease incidence in birds, they cannot completely prevent the infection and shedding of the LPAIV-H9N2 (14, 37).

H9N2 viruses do not induce viremia in infected poultry (38). They suggested that simultaneous viral and bacterial infections influenced more mortality and egg-laying reduction in the respiratory outbreak. It is necessary to develop safe vaccines to conserve against influenza viruses (38). The vaccination program and biosecurity measures are two important tools for preventing and controlling LPAIV-H9N2s in chickens. An experimental formalin-inactivated oil-emulsion H9N2 LPAIV-H9N2 vaccine was reported by Marandi and Fard (38). The prevention of virus shedding through cloaca was employed as the potency test, revealing that the protective doses of 50% (PD_50_) of 1, 1/10, and 1/50 of the field dose of the experimental LPAIV-H9N2 vaccine (EAI) were 100, 100, and 96.25%, respectively. The groups receiving <1/50 dose could not prevent virus shedding. Accordingly, the EAI vaccine could even be entirely protective and efficient in 1/10th dose, leading to a desirable immunity in experimental SPF chickens (39).

Some toxic residues remain in the formalin-inactivated vaccine, and some viruses may escape during chemical inactivation. The typical chemical substances applied for producing inactivated vaccines can cause a reduction in immunogenicity and damage antigenic epitopes (40). γ-Radiation is the perfect method for virus inactivation and the use of ionizing radiation for pathogen inactivation has been developed in the production of effective vaccines (41, 42). γ-Radiation slightly preserves the antigenic construction and can be used in a frozen condition that decreases free radical damage due to water radiolysis (43, 44). Consequently, there are two direct and indirect mechanisms for virus inactivation by γ-irradiation. Direct virus inactivation by γ-irradiation is mainly caused by radiolytic cleavage or cross-linking of genetic materials. The indirect effects of the γ-irradiation stem from the action of free radicals due to the radiolytic cleavage of water. The principal mechanism of virus inactivation by γ-irradiation is damaging viral nucleic acid replication *via* direct and indirect effects (45, 46). One study about LPAIV-H9N2 reported that the virus titer gradually decreased after increasing the dose of γ-radiation (20). The D_10_ value and optimum dose of virus inactivation for LPAIV-H9N2 were calculated by a dose/response curve of 3.36 and 30 kGy, respectively. In addition, the HA antigenicity of γ-irradiated LPAIV-H9N2 subtype H9N2 samples from 0 to 30 kGy represented no change. The safety test for γ-irradiated LPAIV-H9N2 *via* four blind cultures on embryonated eggs showed complete inactivation with γ-ray doses of 30 kGy without any multiplication on the embryonated eggs (20).

Likewise, the γ-irradiation Influenza virus, γ-APC [A/Port Chalmers/1/73(H3N2)], has major immunogenicity, and its protection was 100%, indicating lower weight loss in mice when compared with formalin- or UV-inactivated vaccines (47). Based on their data, γ-ray inactivated virus-induced immunity with high quantitatively and qualitatively to virus preparations inactivated by formalin or UV-irradiation. They further reported that γ-A/PC-vaccinated mice had reduced lung inflammation and viral lung load (47).

This research focused on evaluating both immune responses (humoral and cellular immunity) of the vaccinated broiler chicken in two vaccination regimes (early and late). The HI antibody titration and the proliferation of stimulated spleen lymphocytes had the most upregulation in the IVT and IV+ISA70 vaccine groups at the late vaccination regime 2 weeks after the second vaccination. The first chicken experiment showed the increase of the HI antibody titration and stimulation index of stimulated spleen lymphocytes in IVT.IN compared to the IV.IN vaccine group. These findings suggest that the use of γ-rays preferentially targets the viral genome and has little effect on the functional properties of viral proteins and they could indicate characteristics of Trehalose as a protein stabilizer and protein denaturation inhibitor.

Furthermore, the obtained data revealed that cellular immunity induction and cytokine mRNA expression were upregulated more in the irradiated vaccine groups compared to formalin-treated vaccine groups. IL-2 has been studied widely as a vaccine adjuvant and immuno-enhancer because of its role in activating T-cell proliferation (48). Chicken IFN-γ acts as a cytokine with multiple functions and is primarily secreted by T lymphocytes and NK cells. Further, it can modulate macrophage activation in birds, inhibit viral replication, and develop the Th1 response (49). Th1 cytokines such as IFN-γ and IL-2 were expressed more in the vaccinated chicken groups at the late vaccination age. IL-6 is a protein that is produced by various cells. It helps regulate immune responses, making the IL-6 test potentially useful as a marker of immune system activation. Furthermore, IL-6 is mainly made by some T lymphocytes. It causes B lymphocytes to produce more antibodies and causes fever by affecting the areas of the brain that control body temperature. Moreover, this protein is responsible for stimulating acute phase protein synthesis and producing neutrophils in the bone marrow. Moreover, it supports the growth of B cells and is antagonistic to regulatory T cells (50–52). IL-6 is a pleiotropic cytokine acting as a pro-inflammatory cytokine and an anti-inflammatory myokine. In this study, upregulation of IL-6 was more considerable in the early vaccination than in the late regime. Also, IL-6 was down-regulated in all vaccinated chicken groups in early and late regimes before the challenge with the live virus compared to these groups after the challenge. So, it indicates treatment with live LPAIV-H9N2 virus can upregulate IL-6 more than treatment with inactivated LPAIV-H9N2 virus. The lack of virus shedding in the immunized chicken with irradiated LPAIV-H9N2 and formalin-treated LPAIV-H9N2 vaccines after the challenge indicated a protective response against the live homologous virus. In addition, the viral load of <10^0.5^ TCID_50_/ml in the vaccinated chicken confirmed the protective response for all vaccines in this study. Thus, the IVT vaccine can be considered a good candidate for the immunization of broiler chicken *via* the intranasal route at the late regime. The adaptive immune defenses of newly hatched chickens have limited capabilities to control the pathogens (53). Eventually, Bochen reported that the lowest levels of immunity basically occurred from days 6 to 13 in broiler chickens (54). Accordingly, days 11 and 25 can be recommended as the suitable ages of vaccination against LPAIV-H9N2 for inducing the protective response.

One conclusion of this research is that the use of Trehalose as a protein stabilizer during gamma irradiation can prevent viral antigenic damage and is useful for the production of inactivated vaccine with intact immunogenic characteristics. Another conclusion is that two vaccination doses of irradiated LPAIV-H9N2 vaccine induce more immune responses than one dose of vaccination. Also, the late vaccination regime induces higher immune responses than the early regime. Finally, we can suggest IVT.IN vaccine in the late regime as the suitable vaccine and comfort to inoculation against IV subtype H9N2.

## Data Availability Statement

The datasets presented in this study can be found in online repositories. The names of the repository/repositories and accession number(s) can be found in the article/supplementary material.

## Ethics Statement

The animal study was reviewed and all institutional and national guidelines which were adopted from the horizontal legislation on the protection of animals used for scientific purposes (Directive 2010/63/EU as amended by Regulation (EU) 2019/1010) were approved for implementation Tehran University of Medical Science. Animal trial license number granted by the Tehran University is “IR.TUMS.AEC.1401”.

## Author Contributions

FM, VW, and HU designed the study and helped in interpreting the study results. FM, VW, and SM critically revised the manuscript. IK, AA, and SH carried out the methodology, collected, and analyzed the data. PS and MB have helped in chicken studies and sampling. All authors contributed to the article and approved the submitted version.

## Funding

This study was supported by the International Atomic Energy Agency (IAEA Coordinated Research Project, CRP No. 22126).

## Conflict of Interest

The authors declare that the research was conducted in the absence of any commercial or financial relationships that could be construed as a potential conflict of interest.

## Publisher's Note

All claims expressed in this article are solely those of the authors and do not necessarily represent those of their affiliated organizations, or those of the publisher, the editors and the reviewers. Any product that may be evaluated in this article, or claim that may be made by its manufacturer, is not guaranteed or endorsed by the publisher.
